# Incidence and Risk Factors of Postpartum Hemorrhage in China: A Multicenter Retrospective Study

**DOI:** 10.3389/fmed.2021.673500

**Published:** 2021-08-23

**Authors:** Sijian Li, Jinsong Gao, Juntao Liu, Jing Hu, Xiaoxu Chen, Jing He, Yabing Tang, Xinghui Liu, Yinli Cao, Xiaowei Liu, Xietong Wang

**Affiliations:** ^1^Department of Obstetrics and Gynecology, Peking Union Medical College Hospital, Chinese Academy of Medical Sciences, Peking Union Medical College, National Clinical Research Center for Obstetric and Gynecologic Diseases, Beijing, China; ^2^Department of Obstetrics and Gynecology, Women's Hospital, School of Medicine, Zhejiang University, Zhejiang, China; ^3^Department of Obstetrics and Gynecology, Hunan Maternal and Child Health Care Hospital, Changsha, China; ^4^Department of Obstetrics and Gynecology, Sichuan University West China Second Hospital, Chengdu, China; ^5^Department of Obstetrics and Gynecology, Northwest Women and Children's Hospital, Xi'an, China; ^6^Department of Obstetrics and Gynecology, Beijing Obstetrics and Gynecology Hospital, Capital Medical University, Beijing, China; ^7^Department of Obstetrics and Gynecology, Shandong Provincial Hospital Affiliated to Shandong University, Jinan, China

**Keywords:** postpartum hemorrhage, incidence, risk factors, pregnancy, Chinese population

## Abstract

**Background:** Postpartum hemorrhage (PPH) is a leading cause of maternal morbidity and mortality worldwide but the incidence and its risk factors in China is limited. The objective of this study is to investigate the incidence and the risk factors of PPH in Chinese women.

**Methods:** A multi-center retrospective study of pregnant women at ≥28 weeks of gestation was conducted. Logistic regression was used to identify potential risk factors of PPH and receiver operating characteristic curve was used to evaluate the predictive performance of the identified risk factors. Subgroup analysis focusing on the number of fetus and the mode of delivery was conducted.

**Results:** A total of 99,253 pregnant women were enrolled and 804 (0.81%) experienced PPH. The subgroup analysis revealed that the incidence of PPH was 0.75, 2.65, 1.40, and 0.31% in singletons, twin pregnancies, cesarean sections, and vaginal deliveries, respectively. Placenta previa and placenta accreta were the predominant risk factors of PPH in the overall population and all subgroups. A twin pregnancy was a risk factor for PPH regardless of the mode of delivery. Obesity, and multiparity were risk factors for PPH in both singletons and cesarean section cases, but the latter predicted a reduced probability of PPH in vaginal deliveries. Macrosomia was associated with increased risk of PPH in singletons or vaginal deliveries. In women who delivered vaginally, preeclampsia was associated with a higher risk of PPH. The areas under the curve for the overall cohort, singletons, twin pregnancies, cesarean section cases, and vaginal deliveries were 0.832 (95% confidence interval [CI] 0.813–0.851), 0.824 (95% CI 0.803–0.845), 0.686 (95% CI 0.617–0.755), 0.854 (95% CI 0.834–0.874), and 0.690 (95% CI 0.646–0.735), respectively.

**Conclusions:** The risk factors of PPH varied slightly based on the number of fetuses and the mode of delivery, while placenta previa and placenta accreta were the two major risk factors. A combination of the identified risk factors yielded a satisfactory predictive performance in determining PPH in the overall cohort, singletons pregnancies, and women who delivered by cesarean section, whereas the performance was moderate in twin pregnancies and in women delivering vaginally.

## Introduction

Postpartum hemorrhage (PPH) is the leading cause of maternal mortality worldwide, accounting for approximately 25% of maternal deaths (1, 2). According to the new classifications of the American College of Obstetricians and Gynecologists, it is defined as blood loss exceeding 1,000 mL within 24 h after delivery, regardless of the mode of delivery (3). According to previous nationwide studies, the estimated incidence of PPH ranges from 2.8 to 7.9%, and current researches suggest an increasing trend in PPH (4–8). In Canada, from 2001 to 2009, the incidence of PPH increased from 6.0 to 7.9% and from 1.4 to 2.7%, in vaginal deliveries and cesarean section cases, respectively (4). A nationwide study from the US also revealed that the incidence of PPH increased from 2.9 to 3.2% from 2010 to 2014 (9). Research from other countries also revealed similar results (5).

Conversely, the maternal mortality rate has declined in recent years (10). Nevertheless, it is reported that there are at least 10 “maternal near-misses” for every maternal death due to PPH, including multi-organ dysfunction, multiple blood transfusion, and peripartum hysterectomy (11). Therefore, the proper identification of women at higher risk of PPH is crucial to enable optimization of the available interventions to reduce the associated maternal deaths or other adverse maternal outcomes. Published studies have identified risk factors associated with PPH, including previous history of PPH, preeclampsia, prolonged labor, operative vaginal deliveries, and cesarean section (7, 12–17). However, the large-scale or nationwide studies mainly originate in western countries, and race may also impact the incidence of PPH (18). Results from a Chinese population reported a significant difference in the incidence of PPH, ranging from 2.88 to 15.4% (19, 20). Moreover, previous studies seldom evaluated the predictive performance of these identified risk factors.

Nationwide data on the incidence of PPH among Chinese women are limited, and few studies have evaluated the risk factors for PPH in this large population. We aimed to evaluate the incidence of PPH and investigate the potential risk factors and their predictive performance in a Chinese population.

## Materials and Methods

### Data Sources, Inclusion and Exclusion Criteria, and Definitions

This study was conducted in 14 representative medical centers (including 2 secondary and 12 tertiary hospitals, among them there were 7 general hospitals, and 7 maternal and child health care centers) from 10 provinces in the four major economic regions of China from October 1, 2016, to September 30, 2017. Complete medical information for each birth, including maternal demographics, medical and obstetric history, and maternal and perinatal outcome information was retrospectively registered into a prospectively designed network-based standardized data acquisition system depending on digital and written forms of medical records. This retrospective study was approved by the Ethics Committee of Peking Union Medical College Hospital (approval number: PUMCH-JS-1151). The need for informed consent was waived due to the retrospective nature of the study. All pregnant women aged 18–55years, who had the first antenatal visit in the first trimester and gave birth to fetuses ≥28weeks of gestation were eligible for inclusion. Women with stillbirth, or without data on the exact age and gestational age at delivery were excluded. Data including demographic, medical, and obstetric factors were collected from the medical records and confirmed by the treating physicians.

The main outcome of this study was estimated blood loss ≥1,000 mL within 24 h postpartum. The blood loss volume was visually estimated based on the scaled suction canisters that collected fluids from the surgical field, the area that drenched with blood in surgical drapes, and the number of medical gauzes sodden with blood. The total of blood loss volume was documented in the surgical and nursing records. The demographic factors were maternal age, maternal height, parity, and pre-pregnancy body mass index (BMI). Maternal age was categorized into three groups: <25, 25–34 (reference), ≥35 years. Parity was grouped into nullipara (no previous delivery ≥28 weeks of gestation, reference) and multipara. Maternal height was categorized into three groups: <160 cm, 160–169.9 cm (reference), and ≥ 170 cm. Pre-pregnancy BMI was categorized as underweight (BMI <18.5 kg/m^2^), normal (18.5–23.99 kg/m^2^, reference), overweight (24–27.99 kg/m^2^), and obese (BMI ≥ 28 kg/m^2^) according to the Chinese BMI categorization (21). Medical and obstetric factors refer to methods of conception, number of fetuses [singleton (reference) or twin], hypertensive disorders of pregnancy (HDP), placenta previa (yes or no), placenta accreta (yes or no), mode of delivery [vaginal delivery (reference) or cesarean section], and macrosomia (yes or no). The methods of conception were categorized into natural conception (reference) or assisted reproductive technology (ART). Hypertensive disorders of pregnancy were classified according to the International Society for the Study of Hypertension in Pregnancy guidelines (22). Placenta previa was defined as implantation of the placenta over or very near (<2 cm on ultrasound) to the internal cervical OS. The placenta accreta spectrum, including placenta accreta, increta, and percreta, was defined as placental invasion of the uterine wall based on ultrasound before delivery and verified according to medical records or postnatal placental pathology. Macrosomia was defined as a birth weight of ≥4000g. The exploratory risk factors were maternal age, parity, methods of conception, the number of fetuses, maternal height, pre-pregnancy BMI, mode of delivery, HDP, placenta previa, placenta accreta, and macrosomia.

### Statistical Analysis

Continuous variables are described as means ± standard deviation (range) if they were normally distributed or as medians and interquartile ranges (IQRs) if they were non-normally distributed. Discrete variables are expressed as counts (percentage). Variables were compared between two outcomes (PPH or non-PPH) using univariate analysis. Continuous variables were compared using Student's *t*-test or the Mann–Whitney U-test, depending on their distribution. Categorical variables were compared by the chi-squared test or Fisher's exact test. Variables in the univariate analysis with *P* values <0.2 were selected for potential inclusion in the multivariate logistic regression. Forward, stepwise logistic regression was performed to determine the potential risk factors for PPH. Odds ratios (OR) with 95% confidence intervals (CI) and *P* values were calculated. Area under the receiver operating characteristic (ROC) curve (AUC) was used to evaluate the predictive performance of the identified risk factors. A two-tailed *P* value < 0.05 was considered statistically significant. Statistical analysis was conducted using SPSS (Version 21.0; SPSS Inc., Chicago, IL, USA) or GraphPad prism (Version 8.0) software.

## Results

### Maternal Clinical Characteristics and Incidence of PPH During Pregnancies

A total of 99,253 women met the inclusion criteria and were enrolled in the study, comprising 95,967 singletons and 3,286 twin pregnancies. The mean age at the time of delivery was 30.7 ± 4.5 years, with a mean height of 161.3 ± 4.7 cm. Most pregnancies (96.6%) were naturally conceived and the remaining 3.4% were conceived using ART. The percentages of nulliparous and multiparous women were 58.8 and 41.2%, respectively. Similarly, vaginal delivery accounted for 54.2% of the cohort, whereas deliveries by cesarean section accounted for 45.8%. Only 2.4% of the women were classified as obese.

In this cohort, 804 (0.81%) experienced PPH. Subgroup analysis revealed that the incidence of PPH was 0.75, 2.65, 1.40, and 0.31% in singletons, twin pregnancies, cesarean section cases, and vaginal deliveries, respectively. Meanwhile 1,871 (3.48%) women experienced blood loss ≥ 500 mL in vaginal deliveries. The incidence of HDP was 5.1%, with a rate of 2.9 and 0.3% for preeclampsia and preeclampsia superimposed on chronic hypertension, respectively. Both categories were classified as preeclampsia in our study to evaluate the effect on PPH. The detailed characteristics and obstetric outcomes are listed in Table 1.

**Table 1:** Maternal clinical characteristics and outcomes in 99253 pregnant women.

**Total**	***N*** **= 99253**	**Percentile**
Singleton	95967	96.69%
Twin	3286	3.31%
Age (y)	30.7 ± 4.5	
<25	6373	6.4%
25–34	72356	72.9%
≥35	20524	20.7%
Parity		
Nulli	58336	58.8%
Pluri (parity 1–3 times)	40535	40.7%
Pluri (parity ≥ 4 times)	382	0.4%
Gestational age (weeks)	38.8 ± 2.0	
≥37	88563	89.2%
34-36w+6d	7172	7.2%
32-33w+6d	1672	1.7%
<32	1846	1.9%
Methods of conception		
Nature	95893	96.6%
ART	3360	3.4%
Height (cm)	161.3 ± 4.7	
<160	33195	62.0%
160–169.9	61535	33.4%
≥170	4523	4.6%
Pre-pregnancy BMI (kg/m^2^)	20.9 ± 3.1	
<18.5	22687	23.5%
18.5–23.9	61085	63.3%
24.0–27.9	10427	10.8%
≥28.0	2362	2.4%
Mode of delivery		
Vaginal delivery	53798	54.2%
Cesarean section	45455	45.8%
Cesarean section history	18433	18.6%
Hypertensive disorders of pregnancy	5095	5.1%
Gestation hypertension	1853	1.9%
Chronic hypertension	150	0.2%
Preeclampsia	2803	2.9%
Preeclampsia superimposed		
On chronic hypertension	289	0.3%
Postpartum hemorrhage		
≥1,000 ml (overall population)	804	0.8%
Singletons pregnancies subgroup	717	0.75%
Twin pregnancies subgroup	87	2.65%
Cesarean delivery subgroup	637	1.40%
Vaginal delivery subgroup	167	0.31%

### Risk Factors of PPH in This Cohort and Subgroups

The potential risk factors were evaluated in univariate and multivariate logistic regression analyses (Table 2, Supplementary Table 1). All these exploratory factors appeared to be associated with PPH in the univariate analysis. However, subsequent multivariate analysis revealed that multiparity (OR 1.299, 95% CI 1.110–1.521, *P* = 0.001), twin pregnancy (OR 3.487, 95% CI 2.677–4.543, *P* < 0.001), obesity (pre-pregnancy body mass index ≥ 28 kg/m^2^) (OR 1.821, 95% CI 1.245–2.662, *P* = 0.002), cesarean section (OR 1.407, 95% CI 1.149–1.723, *P* = 0.001), placenta previa (OR 13.394, 95% CI 11.033–16.260, *P* < 0.001), placenta accreta (OR 9.697, 95% CI 8.051–11.679, *P* < 0.001), and macrosomia (OR 1.863, 95% CI 1.391–2.494, *P* < 0.001) were the risk factors of PPH in the overall cohort.

**Table 2:** Adjusted OR and AUC in the predictive model for PPH in pregnant women (*N* = 99,253).

**Variables**	**Multivariate logistic regression**			**AUC** **(95% CI)**	***P*** **value**
	**Adjusted OR**	**95% CI**	***P*** **value**		
Parity				0.581 (0.561–0.601)	<0.001
Nulli		Ref.			
Pluri	1.299	1.110–1.521	0.001		
Group gestation				0.539 (0.517–0.560)	<0.001
Singleton		Ref.			
Twin	3.487	2.677–4.543	<0.001		
Pre-pregnancy BMI (kg/m^2^)			<0.001	0.518 (0.496–0.539)	0.087
<18.5	0.871	0.708–1.072	0.194		
18.5–23.9		Ref.			
24.0–27.9	1.286	1.036–1.595	0.022		
≥28.0	1.821	1.245–2.662	0.002		
Mode of delivery				0.670 (0.653–0.687)	<0.001
Vaginal dellivery		Ref.			
Cesarean section	1.407	1.149–1.723	0.001		
Placenta previa				0.731 (0.709–0.754)	<0.001
No		Ref.			
Yes	13.394	11.033–16.260	<0.001		
Placenta accreta				0.727 (0.705–0.750)	<0.001
No		Ref.			
Yes	9.697	8.051–11.679	<0.001		
Macrosomia				0.508 (0.488–0.529)	0.437
No		Ref.			
Yes	1.863	1.391–2.494	<0.001		

A subgroup analysis involving the number of fetuses and the mode of delivery was conducted. The results revealed that the risk factors differed between singletons and twin gestations. In singletons, maternal age and height, parity, methods of conception, mode of delivery, pre-pregnancy BMI, placenta previa, placenta accreta, and macrosomia were likely associated with PPH and were assigned to multivariate logistic regression (Table 3, Supplementary Table 2). Multiparity (OR 1.342, 95% CI 1.134–1.587, *P* = 0.001), overweight (OR 1.422, 95% CI 1.136–1.780, *P* = 0.002) or obesity (OR 2.047, 95% CI 1.480–3.037, *P* < 0.001), cesarean section (1.353, 95% CI 1.096–1.670, *P* = 0.005), placenta previa (OR 14.432, 95% CI 11.742–17.738, *P* < 0.001), placental accreta (OR 10.079, 95% CI 8.263–12.296, *P* < 0.001), and macrosomia (OR 1.893, 95% CI 1.407–2.547, *P* < 0.001) predicted a higher risk of PPH in singletons (Table 3). In twin pregnancies, maternal age (*P* = 0.049), methods of conception (*P* = 0.044), pre-pregnancy BMI (*P* = 0.088), HDP (*P* = 0.167), placenta previa (*P* < 0.001), and placenta accreta (*P* < 0.001) were included in the multivariate logistic model. However, only placenta previa (OR 5.898, 95% CI, 3.130–11.113, *P* < 0.001) and placenta accreta (OR 6.694, 95% CI 3.905–11.474, *P* < 0.001) remained statistically significant (Table 4, Supplementary Table 3).

**Table 3:** Adjusted OR and AUC in the predictive model for PPH in singleton pregnancies (*N* = 95,967).

**Variables**	**Multivariate logistic regression**			**AUC** **(95% CI)**	***P*** **value**
	**Adjusted OR**	**95% CI**	***P*** **value**		
Parity				0.595 (0.574–0.616)	<0.001
Nulli		Ref.			
Pluri	1.342	1.134–1.587	0.001		
Pre-pregnancy BMI (kg/m^2^)			<0.001	0.528 (0.505–0.551)	0.011
<18.5	0.874	0.701–1.090	0.231		
18.5–23.9		Ref.			
24.0–27.9	1.422	1.136–1.780	0.002		
≥28.0	2.047	1.380–3.037	<0.001		
Mode of delivery				0.670 (0.651–0.688)	<0.001
Vaginal dellivery		Ref.			
Cesarean section	1.353	1.096–1.670	0.005		
Placenta previa				0.745 (0.722–0.769)	<0.001
No		Ref.			
Yes	14.432	11.742–17.738	<0.001		
Placenta accreta				0.734 (0.711–0.758)	<0.001
No		Ref.			
Yes	10.079	8.263–12.296	<0.001		
Macrosomia				0.511 (0.489–0.533)	0.315
No		Ref.			
Yes	1.893	1.407–2.547	<0.001		

**Table 4:** Adjusted OR and AUC in the predictive model for PPH in twin pregnancies (*N* = 3,286).

**Variables**	**Multivariate logistic regression**			**AUC** **(95% CI)**	***P*** **value**
	**Adjusted OR**	**95% CI**	***P*** **value**		
Placenta previa				0.615 (0.546–0.684)	<0.001
No		Ref.			
Yes	5.898	3.130–11.113	<0.001		
Placenta accreta				0.660 (0.591–0.729)	<0.001
No		Ref.			
Yes	6.694	3.905–11.474	<0.001		

The risk factors of PPH varied in the subgroups based on the mode of delivery. In patients who underwent cesarean section, maternal age, parity, methods of conception, the number of fetuses, pre-pregnancy BMI, placenta previa, and placenta accreta were significantly associated with PPH in the univariate analysis (*P* < 0.01) (Supplementary Table 4). In subsequent multivariate regression, multiparity (OR 1.562, 95% CI 1.298–1.878, *P* < 0.001), twin pregnancy (OR 3.227, 95% CI 2.451–4.249, *P* < 0.001), obesity (OR 1.794, 95% CI 1.176–2.736, *P* = 0.007), placenta previa (OR 12.332, 95% CI 10.048–15.135, *P* < 0.001), and placenta accreta (OR 9.573, 95% CI 7.822–11.717, *P* < 0.001) predicted a higher risk of PPH (Table 5). In women undergoing vaginal deliveries, factors that may affect preterm birth are summarized in Supplementary Table 5. ART, twin pregnancy, HDP, placenta previa, placenta accreta, and macrosomia were associated with PPH in the univariate analysis (*P* < 0.01). Maternal age (*P* = 0.072), parity (*P* = 0.047), and pre-pregnancy BMI (*P* = 0.039) seemed to affect the risk of PPH. These factors were further used in a multivariate analysis, whereby twin pregnancies (OR 6.682, 95% CI 2.902–15.338, *P* < 0.001), preeclampsia or more severe HDP (OR 3.866, 95% CI 1.575–9.485, *P* = 0.003), placenta previa (OR 20.367, 95% CI 11.236–36.918, *P* < 0.001), placenta accreta (OR 10.674, 6.504–17.518, *P* < 0.001), and macrosomia (OR 2.737, 95% CI 1.662–4.506, *P* < 0.001) were risk factors for PPH among patients with vaginal deliveries (Table 6). However, multiparity was associated with a significantly decreased risk for PPH (OR 0.647, 95% CI 0.452–0.925, *P* = 0.017).

**Table 5:** Adjusted OR and AUC in the predictive model for PPH in cesarean section cases (*N* = 45,455).

**Variables**	**Multivariate logistic regression**			**AUC** **(95% CI)**	***P*** **value**
	**Adjusted OR**	**95% CI**	***P*** **value**		
Parity				0.570 (0.548–0.592)	<0.001
Nulli		Ref.			
Pluri	1.562	1.298–1.878	<0.001		
Group gestation				0.531 (0.507–0.555)	0.007
singleton		Ref.			
Twin	3.227	2.451–4.249	<0.001		
Pre-pregnancy BMI (kg/m^2^)			0.003	0.510 (0.486–0.534)	0.394
<18.5	0.868	0.680–1.109	0.258		
18.5–23.9		Ref.			
24.0–27.9	1.301	1.024–1.654	0.031		
≥28.0	1.794	1.176–2.736	0.007		
Placenta previa				0.773 (0.750–0.797)	<0.001
No		Ref.			
Yes	12.332	10.048–15.135	<0.001		
Placenta accreta				0.766 (0.742–0.790)	<0.001
No		Ref.			
Yes	9.573	7.822–11.717	<0.001		

**Table 6:** Adjusted OR and AUC in the predictive model for PPH in vaginal deliveries (*N* = 53,798).

**Variables**	**Multivariate logistic regression**			**AUC** **(95% CI)**	***P*** **value**
	**Adjusted OR**	**95% CI**	***P*** **value**		
Parity				0.463 (0.421–0.506)	0.100
Nulli		Ref.			
Pluri	0.647	0.452–0.925	0.017		
Group gestation				0.518 (0.473–0.564)	0.413
Singleton		Ref.			
Twin	6.682	2.902–15.388	<0.001		
HDP			0.002	0.525 (0.479–0.571)	0.263
No		Ref.			
GH or cHTN	2.429	1.058–5.580	0.036		
PE	3.866	1.575–9.485	0.003		
Placenta previa				0.540 (0.493–0.587)	0.075
No		Ref.			
Yes	20.367	11.236–36.918	<0.001		
Placenta accreta				0.554 (0.507–0.602)	0.015
No		Ref.			
Yes	10.674	6.504–17.518	<0.001		
Macrosomia				0.531 (0.485–0.577)	0.162
No		Ref.			
Yes	2.737	1.662–4.506	<0.001		

*ART, assistant reproductive technology; BMI, body mass index; OR, odds ratio; AUC, area under the curve; CI, confidence interval; Ref., reference; PPH, postpartum hemorrhage; HDP, hypertensive disorders of pregnancy; cHTN, chronic hypertension; GH, gestational hypertension; PE, preeclampsia*.

### Predictive Performance of the Identified Risk Factors for PPH

**Figure 1:**
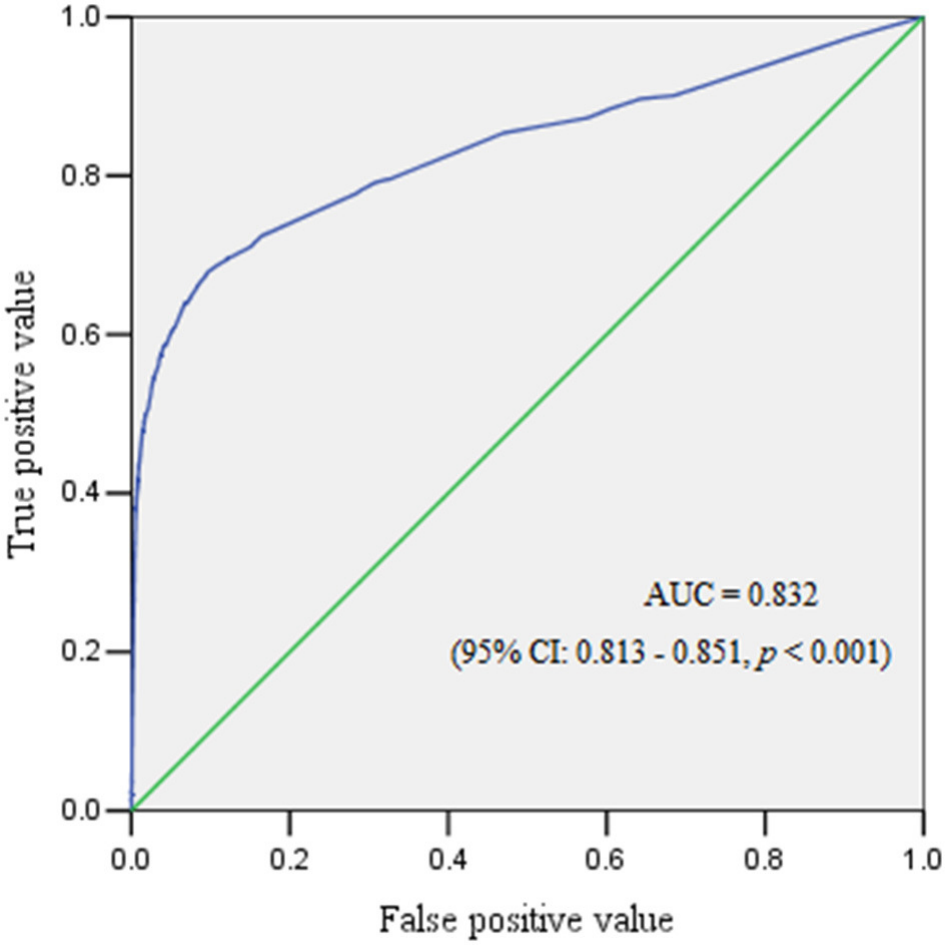
The receiver operating characteristic curve demonstrated the predictive performance for postpartum hemorrhage.

**Figure 2:**
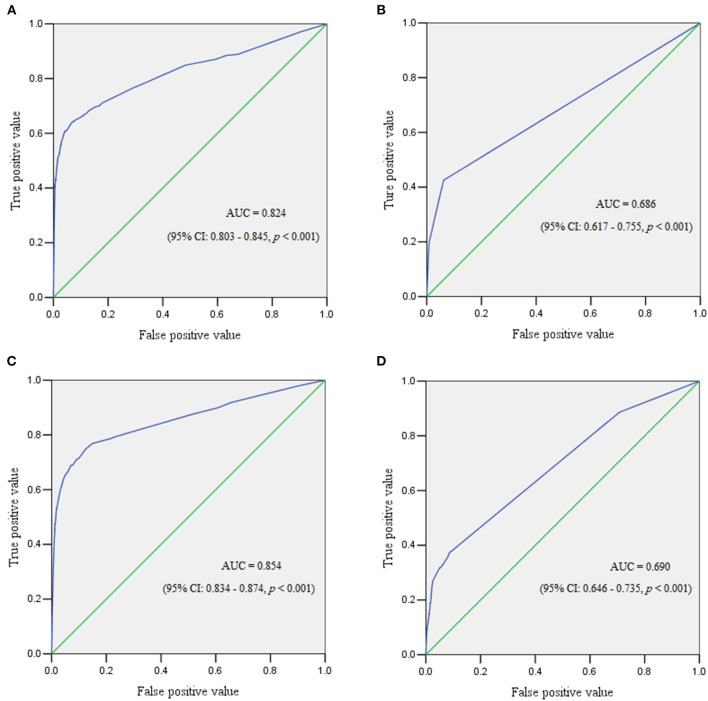
The receiver operating characteristic curve demonstrated the predictive performance for postpartum hemorrhage in subgroups. **(A)** Receiver operating characteristic curve for singleton pregnancies. **(B)** Receiver operating characteristic curve for twin pregnancies. **(C)** Receiver operating characteristic curve for women who underwent cesarean section. **(D)** Receiver operating characteristic curve for women with vaginal deliveries.

The ROC curve reflected the predictive performance of the model using single variable or combined risk factors for PPH (Tables 2–6; Figures 1, 2). In the predictive model for this cohort and all the subgroups, placenta previa and placenta accreta were two factors that had the highest AUC in the single variable model. Meanwhile, the performance of the other factors was relatively unsatisfactory, with an AUC between 0.5 and 0.6. Moreover, in the subgroup analysis, the predictive performance was better in singletons and cesarean section cases, regardless of whether single variable predictive models or combination predictive models were used. Moreover, a combination of these identified risk factors can significantly increase the predictive performance in the overall cohort as well as in the subgroups. The AUC of the combination of these identified risk factors for the overall cohort, singletons, twin pregnancies, cesarean section cases, and vaginal deliveries was 0.832 (95% CI 0.813–0.851), 0.824 (95% CI 0.803–0.845), 0.686 (95% CI 0.617–0.755), 0.854 (95% CI 0.834–0.874), and 0.690 (95% CI 0.646–0.735), respectively (Figures 1, 2).

## Discussion

Our study presents one of the largest cohorts involving the incidence and risk factors of PPH in Chinese women. The incidence of PPH was relatively low in the overall population and the four specific subgroups. Placenta previa and placenta accreta were the predominant risk factors for PPH in the overall population and in all subgroups, whereas multiparity, twin pregnancies, obesity, macrosomia, cesarean section, and preeclampsia were factors associated with PPH. A combination of the identified risk factors yielded a satisfactory predictive performance for PPH in singletons and women undergoing cesarean section. This research could aid in the risk identification and stratification of PPH, thereby optimizing management in this population.

The incidence of PPH varied in different countries and regions (4–7, 19, 23). A study including 8.5 million pregnant women in the US revealed that the incidence of PPH was 2.8% in 2008 (7). In 2014, another larger study including >30 million patients reported a similar rate of 3.2% (9). In Canada, research in 2009 reported a 7.9% incidence of PPH in vaginal deliveries and 2.7% in cesarean section cases (4). Data in Ireland were similar, with an overall incidence of 4.1% (5). However, investigation of a relatively small sample size from China yielded a significantly higher rate of 15.4 and 3.3% in vaginal deliveries and cesarean section cases, respectively (19). Research conducted in Shanxi province, China, revealed that the PPH rate was 2.88% in 127,145 pregnant women (20). Our study demonstrated an extremely low incidence of PPH compared with previous research, which could be partly explained by the method of estimation of blood loss in our research. The visual estimation of blood loss may underestimate the true blood loss, sometimes by 30–50% (24, 25). The exact incidence of PPH could therefore be higher than our result. Moreover, we defined PPH as blood loss ≥1,000 mL. The overall incidence would be 2.53% if we defined PPH as blood loss ≥500 mL in vaginal deliveries (1,871 cases in 53,798 patients, 3.48%) and blood loss ≥1000 mL in cesarean section cases, which would be comparable with other researches.

Placenta previa and placenta accreta were the two factors that most significantly increased the risk of PPH, by approximately 6-fold to 20-fold risk in the former and 7-fold to 11-fold in the latter, compared to patients without these complications. The overall incidence of PPH was 15.49, and 13.48% in placenta previa and placenta accreta, respectively. This result confirmed the previous finding that women with placenta previa and placenta accreta were at high risk of PPH, which could sometimes be life-threatening. A study in the US revealed that patients with placenta previa had a 7-fold increased risk of severe PPH compared to those with normal placental position (7, 26, 27). In a meta-analysis involving 5,146 pregnant women with placenta previa, the overall incidence of PPH was 22.3%, with the incidence being 27.4 and 14.5% in placenta previa and low-lying placenta previa cases, respectively (28). Meanwhile, this phenomenon in both placenta previa and placenta accreta was more significant in vaginal deliveries than in cesarean section cases (OR 20.367 vs. 12.332, and 10.674 vs. 9.573, respectively). Similar results were also observed by comparing patients with singleton gestations and twin gestations. This result is not surprising, because the guidelines for placenta previa and placenta accreta recommend planned cesarean section by experienced teams or at tertiary centers in most circumstances for better maternal outcomes (26, 27). A study reported that the outcomes were better in patients antenatally diagnosed with placenta accreta that in those unexpectedly diagnosed at the time of delivery, with the estimated blood loss and red blood cell transfusion being significantly higher in the unexpected group [2.4 L (1.4–3) vs. 1.7 L (1.2–3), *P* = 0.04; red blood cell units, 4 (1–6) vs. 2 (0–5), *P* = 0.03] (29). In our study, some patients with a low-lying placenta and unexpected placenta accreta underwent vaginal delivery, while the proportion of twin pregnancies delivered by cesarean section was significantly higher than that of singletons (91.2% vs. 44.2%, *P* < 0.001); vaginal delivery in these situations increased the risk of PPH caused by placenta previa and placenta accreta. These findings confirm the importance of early identification of placenta previa and placenta accreta and the subsequent close monitoring, and planned delivery by experienced teams to improve maternal outcomes.

The impact of other risk factors identified in our study on PPH was relatively mild to moderate. Our study demonstrated twin pregnancy increased a 3 to 6-fold risk of PPH in the overall cohort and cesarean section and vaginal delivery subgroups, which reinforced the awareness of the increased risk of adverse outcomes associated with multiple gestations; therefore, obstetric concerns will increase as the rate of twin pregnancies is increasing (30, 31). Multipara and patients who underwent cesarean section were at higher risk of PPH in the overall cohort and the singleton subgroup, in accordance with previous research (7, 14, 23). Meanwhile, a large proportion of multipara underwent cesarean section due to having undergone a prior cesarean section; repeat cesarean section has also been found to be a risk factor for PPH (20). Besides, a higher parity demonstrated a more pronounced risk on PPH in the overall population, singletons, and cesarean delivery subgroups (Supplementary Table 6), which was consistent with previous study that high parity was associated with uterine atony (4). Conversely, multiparity led to a decreased risk of PPH in patients undergoing vaginal deliveries, which may be attributed to a shorter mean duration of labor and reduced probability of severe perineal laceration compared with that in nullipara; published studies have linked prolonged labor and severe perineal laceration with an increased risk of PPH (15–17, 32). Our study demonstrated a high cesarean delivery rate of 45.8%, which is associated with increased risk of PPH compared to vaginal delivery. Given these findings, it is reasonable to recommend a vaginal delivery if there is no contraindication and unnecessary cesarean section should be avoided to reduce the risk of PPH. Relative strategies to lower the cesarean delivery rate are warranted.

Pre-pregnancy obesity and fetal macrosomia were two risk factors identified in this cohort, in the singleton and vaginal delivery subgroups, but not in twin gestation and cesarean section subgroups. Fetal macrosomia as a risk factor for predicting PPH has been confirmed in previous studies (15, 17, 33). Recently, a meta-analysis involving 350,311 pregnancies revealed that fetal macrosomia was associated with a higher risk of prolonged first and second stage of labor (OR 1.57; 95% CI 1.51–1.63 and OR 2.03; 95% CI 1.88–2.19, respectively), an increased risk of instrumental vaginal delivery (OR 1.76; 95% CI 1.68–1.85), third degree perineal trauma (OR 2.73; 95% CI 2.30–3.23), and emergency cesarean section (OR 1.84; 95% CI 1.75–1.93), and these factors were also risk factors for PPH (34). Maternal pre-pregnancy obesity has been found to be positively correlated with large for gestational age (1.88, 95% CI 1.67–2.11) or fetal macrosomia (OR 2.92, 95% CI 2.67–3.20) (35), and to be associated with a higher likelihood of having gestational diabetes mellitus (36), which in turn contributes to fetal macrosomia. These have a synergistic effect and significantly increase the risk of PPH. Nonetheless, being underweight pre-pregnancy is also associated with adverse perinatal outcomes such as preterm birth and delivering an infant small for gestational age (35); a normal pre-pregnancy BMI and accurate estimation of fetal weight to reduce the chance of macrosomia, instrumental vaginal delivery, and emergency cesarean section are advisable.

Preeclampsia impacted the risk of PPH in patients who underwent vaginal delivery in our study; this effect was absent in the other subgroups. Moreover, the incidence of PPH exhibited no significant difference between patients with gestational hypertension or chronic hypertension complicated pregnancies and those without HDP. This suggests that mild HDP may not be associated with an increased risk of PPH. Endothelial dysfunction, impaired uteroplacental blood flow, coagulation abnormality can be found in severe HDP, but these pathological changes may not be found in mild HDP (37). Although other research also identified preeclampsia as a risk factor for PPH, but the underlying mechanisms remain uncertain (7, 12). Some studies investigated the relationship between labor progression and HDP but they yielded inconsistent results (38, 39). Nonetheless, since preeclampsia is also associated with more frequent adverse maternal and fetal outcomes in addition to PPH (37), the importance of prevention and effective control of HDP should be underscored.

Risk assessment tools are available, which can identify 60–85% of patients who will experience a significant obstetric hemorrhage, as reported in previous studies (7, 40), but the specificity was relatively low at just below 60% in a large validation study (40). Another study evaluated risk assessment tools for severe PPH in cesarean section cases and showed that the prediction performance was moderate, with an AUC of ~0.7–0.8 (41). A retrospective study created a better predictive model, but the sample size was relatively small because only 2,336 pregnant women were enrolled (42). Our research demonstrated a different predictive performance in this cohort and the four subgroups. Any single risk factor displayed poor performance for predicting PPH, with an AUC of 0.5–0.6, except for placenta previa or placenta accreta (AUC > 0.6 in twins and AUC > 0.7 in other groups). This indicated that in patients with single risk factor, only placenta previa and placenta accreta may be reliable predictors, which may also help to risk stratifications the of PPH. A combination of all the identified risk factors yielded a satisfactory predictive performance in the overall cohort, as well as in the singleton and cesarean section subgroups; but it was moderate in twin pregnancy and vaginal delivery subgroups. Moreover, the true positive value (TPV) was low (~60%) even when the false positive value (FPV) was 40% in twin pregnancy and vaginal delivery subgroups. However, the result was much better in the other three groups, with a TPV of ~80% when the FPV was 20%. This may suggest that the conventionally identified risk factors were not so sensitive, specific, and accurate in predicting PPH. Recently, a study applied machine learning and statistical models to better predict PPH and exhibited excellent discriminative ability in 152,279 assessed births (43). This may assist health care providers to be prepared and effectively triage at-risk women, but further research is needed.

The strength of this study is the large sample size which included participants from multiple centers in different districts in China. Data from the 14 hospitals in the four major economic regions of China provide reliable reference for clinical practice. Meanwhile, the large sample size enabled us to conduct subgroup analysis to evaluate the potential risk factors for PPH based on the number of fetuses and the mode of delivery. However, the study also has some limitations. First, the retrospective nature may affect the validity of the analysis. Second, this study lacks risk factors of severe PPH and long-term follow-up of maternal outcomes in those who experienced PPH. Third, the effect of drug administration during labor on PPH was not evaluated due to a lack of detailed records. Therefore, further studies are required.

## Conclusion

The risk factors of PPH varied slightly based on the number of fetuses and the mode of delivery. Placenta previa and placenta accreta were the two major risk factors for PPH. A combination of the identified risk factors yielded a satisfactory predictive performance in predicting PPH in the overall cohort, singleton pregnancies, and women who delivered by cesarean section, whereas the performance was moderate in twin pregnancies and women who underwent vaginal deliveries.

## Data Availability Statement

The original contributions presented in the study are included in the article/Supplementary Material, further inquiries can be directed to the corresponding author.

## Ethics Statement

This retrospective study was approved by the Ethics Committee of Peking Union Medical College Hospital (reference number: PUMCH-JS-1151).

## Author Contributions

SL wrote the manuscript and participated in data analysis. JHe, YT, XinL, YC, XiaL, and XW conducted data collection and quality control at their medical centers, JHu and XC participated in data analysis. JG conceived and designed the study. JL participated in designing the study and revising the language. All authors read and approved the manuscript.

## Conflict of Interest

The authors declare that the research was conducted in the absence of any commercial or financial relationships that could be construed as a potential conflict of interest.

## Publisher's Note

All claims expressed in this article are solely those of the authors and do not necessarily represent those of their affiliated organizations, or those of the publisher, the editors and the reviewers. Any product that may be evaluated in this article, or claim that may be made by its manufacturer, is not guaranteed or endorsed by the publisher.
