# Supplementary material for: Incidence and Risk Factors of Postpartum Hemorrhage in China: A Multicenter Retrospective Study
Source: Front Med (Lausanne). 2021 Aug 23;8:673500. doi: 10.3389/fmed.2021.673500 (PMC8419315; doi:10.3389/fmed.2021.673500)
Supplement: Supplementary file 1 [file Table_1.DOCX]

Table S1. Logistics regression to identify potential risk factors for PPH (N = 99253).

| Variables | Group Control  (n = 98449) | Group PPH  (n = 804) | P  value | Multivariate logistic regression | | |
| --- | --- | --- | --- | --- | --- | --- |
|  |  |  |  | Adjusted OR | 95% CI | P value |
| Age(y)* |  |  | <0.001 |  |  |  |
| <25 | 6339 (6.4%) | 34 (4.2%) |  |  |  |  |
| 25-34 | 71832 (73.0%) | 524 (65.2%) |  |  |  |  |
| >=35 | 20278 (20.6%) | 246 (30.6%) |  |  |  |  |
| Parity* |  |  | <0.001 |  |  |  |
| Nulli | 57994 (58.9%) | 342 (42.5%) |  |  | Ref. |  |
| Pluri | 40455 (41.1%) | 462 (57.5%) |  | 1.299 | 1.110-1.521 | 0.001 |
| Conception* |  |  | <0.001 |  |  |  |
| Natural | 95162 (96.7%) | 731 (90.9%) |  |  |  |  |
| ART | 3287 (3.3%) | 73 (9.1%) |  |  |  |  |
| Group gestation* |  |  | <0.001 |  |  |  |
| Singleton | 95250 (96.8%) | 717 (89.2%) |  |  | Ref. |  |
| Twin | 3199 (3.2%) | 87 (10.8%) |  | 3.487 | 2.677-4.543 | <0.001 |
| Height (cm)* |  |  | 0.038 |  |  |  |
| < 160 | 32907 (33.4%) | 493 (61.3%) |  |  |  |  |
| 160-169 | 61042 (62.0%) | 288 (35.8%) |  |  |  |  |
| > = 170 | 4500 (4.6%) | 23 (2.9%) |  |  |  |  |
| Pre-pregnancy BMI (kg/m^2^) * |  |  | <0.001 |  |  | <0.001 |
| <18.5 | 22559 (23.6%) | 128 (16.3%) |  | 0.871 | 0.708-1.072 | 0.194 |
| 18.5-23.9 | 60592 (63.3%) | 493 (62.6%) |  |  | Ref. |  |
| 24.0-27.9 | 10297 (10.8%) | 130 (16.5%) |  | 1.286 | 1.036-1.595 | 0.022 |
| >=28.0 | 2326 (2.4%) | 36 (4.6%) |  | 1.821 | 1.245-2.662 | 0.002 |
| Mode of delivery* |  |  | <0.001 |  |  |  |
| Vaginal dellivery | 53631 (54.5%) | 167 (20.8%) |  |  | Ref. |  |
| Cesarean section | 44818 (45.5%) | 637 (79.2%) |  | 1.407 | 1.149-1.723 | 0.001 |
| HDP* |  |  | 0.011 |  |  |  |
| No | 93413 (94.9%) | 745 (92.7%) |  |  |  |  |
| GH or cHTN | 1983 (2.0%) | 20 (2.5%) |  |  |  |  |
| PE | 3053 (3.1%) | 39 (4.9%) |  |  |  |  |
| Placenta previa* |  |  | <0.001 |  |  |  |
| No | 96321 (97.8%) | 414 (51.5%) |  |  | Ref. |  |
| Yes | 2128 (2.2%) | 390 (48.5%) |  | 13.394 | 11.033-16.260 | <0.001 |
| Placenta accrete* |  |  | <0.001 |  |  |  |
| No | 95978 (97.5%) | 419 (52.1%) |  |  | Ref. |  |
| Yes | 2471 (2.5%) | 385 (47.9%) |  | 9.697 | 8.051-11.679 | <0.001 |
| Macrosomia* |  |  | 0.078 |  |  |  |
| No | 92901 (94.4%) | 747 (92.9%) |  |  | Ref. |  |
| Yes | 5548 (5.6%) | 57 (7.1%) |  | 1.863 | 1.391-2.494 | <0.001 |

*Factors assigned to multivariate logistic regression analysis.

Abbreviations: Ref., reference; PPH, postpartum hemorrhage; ART, assistant reproductive technology; BMI, body mass index; HDP, hypertensive disorders of pregnancy; cHTN, chronic hypertension; GH, gestational hypertension; PE, preeclampsia.
