# Supplementary material for: Incidence and Risk Factors of Postpartum Hemorrhage in China: A Multicenter Retrospective Study
Source: Front Med (Lausanne). 2021 Aug 23;8:673500. doi: 10.3389/fmed.2021.673500 (PMC8419315; doi:10.3389/fmed.2021.673500)
Supplement: Supplementary file 2 [file Table_2.DOCX]

Table S2. Logistics regression to identify potential risk factors for PPH in singleton pregnancy (N = 95967).

| Variables | Group Control  (n = 95250) | Group PPH  (n = 717) | P  value | Multivariate logistic regression | | |
| --- | --- | --- | --- | --- | --- | --- |
|  |  |  |  | Adjusted OR | 95% CI | P value |
| Age(y)* |  |  | <0.001 |  |  |  |
| <25 | 6157 (6.5%) | 29 (4.0%) |  |  |  |  |
| 25-34 | 69462 (72.9%) | 469 (65.4%) |  |  |  |  |
| >=35 | 19631 (20.6%) | 219 (30.5%) |  |  |  |  |
| Parity* |  |  | <0.001 |  |  |  |
| Nulli | 55930 (58.7%) | 284 (39.6%) |  |  | Ref. |  |
| Pluri | 39320 (41.3%) | 433 (60.4%) |  | 1.342 | 1.134-1.587 | 0.001 |
| Conception* |  |  | 0.001 |  |  |  |
| Natural | 93190 (97.8%) | 687 (95.8%) |  |  |  |  |
| ART | 2060 (2.2%) | 30 (4.2%) |  |  |  |  |
| Mode of delivery* |  |  | <0.001 |  |  |  |
| Vaginal dellivery | 53348 (56.0%) | 160 (22.3%) |  |  | Ref. |  |
| Cesarean section | 41902 (44.0%) | 557 (77.7%) |  | 1.353 | 1.096-1.670 | 0.005 |
| Height (cm)* |  |  | 0.068 |  |  |  |
| < 160 | 31826 (33.4%) | 259 (36.1%) |  |  |  |  |
| 160-169 | 59036 (62.0%) | 436 (60.8%) |  |  |  |  |
| > = 170 | 4388 (4.6%) | 22 (3.1%) |  |  |  |  |
| Pre-pregnancy BMI (kg/m^2^) * |  |  | <0.001 |  |  | <0.001 |
| <18.5 | 21904 (23.6%) | 115 (16.4%) |  | 0.874 | 0.701-1.090 | 0.231 |
| 18.5-23.9 | 58704 (63.3%) | 428 (61.1%) |  |  | Ref. |  |
| 24.0-27.9 | 9895 (10.7%) | 123 (17.5%) |  | 1.422 | 1.136-1.780 | 0.002 |
| >=28.0 | 2219 (2.4%) | 35 (5.0%) |  | 2.047 | 1.380-3.037 | <0.001 |
| HDP |  |  | 0.368 |  |  |  |
| No | 90614 (95.1%) | 674 (94.0%) |  |  |  |  |
| GH or cHTN | 1874 (2.0%) | 17 (2.4%) |  |  |  |  |
| PE | 2762 (2.9%) | 26 (3.6%) |  |  |  |  |
| Placenta previa* |  |  | <0.001 |  |  |  |
| No | 93193 (97.8%) | 349 (48.7%) |  |  | Ref. |  |
| Yes | 2057 (2.2%) | 368 (51.3%) |  | 14.432 | 11.742-17.738 | <0.001 |
| Placenta accrete* |  |  | <0.001 |  |  |  |
| No | 92932 (97.6%) | 364 (50.8%) |  |  | Ref. |  |
| Yes | 2318 (2.4%) | 353 (49.2%) |  | 10.079 | 8.263-12.296 | <0.001 |
| Macrosomia* |  |  | 0.025 |  |  |  |
| No | 89737 (94.2%) | 661 (92.2%) |  |  | Ref. |  |
| Yes | 5513 (5.8%) | 56 (7.8%) |  | 1.893 | 1.407-2.547 | <0.001 |

*Factors assigned to multivariate logistic regression analysis.

Abbreviations: Ref., reference; PPH, postpartum hemorrhage; ART, assistant reproductive technology; BMI, body mass index; HDP, hypertensive disorders of pregnancy; cHTN, chronic hypertension; GH, gestational hypertension; PE, preeclampsia.
