# Supplementary material for: Incidence and Risk Factors of Postpartum Hemorrhage in China: A Multicenter Retrospective Study
Source: Front Med (Lausanne). 2021 Aug 23;8:673500. doi: 10.3389/fmed.2021.673500 (PMC8419315; doi:10.3389/fmed.2021.673500)
Supplement: Supplementary file 3 [file Table_3.DOCX]

Table S3. Logistics regression to identify potential risk factors for PPH in twin pregnancy (N = 3286).

| Variables | Group Control  (n = 3199) | Group PPH  (n = 87) | P  value | Multivariate logistic regression | | |
| --- | --- | --- | --- | --- | --- | --- |
|  |  |  |  | Adjusted OR | 95% CI | P value |
| Age(y)* |  |  | 0.049 |  |  |  |
| <25 | 182 (5.7%) | 5 (4.0%) |  |  |  |  |
| 25-34 | 2370 (74.1%) | 55 (65.4%) |  |  |  |  |
| >=35 | 647 (20.2%) | 27 (30.5%) |  |  |  |  |
| Parity |  |  | 0.734 |  |  |  |
| Nulli | 2064 (64.5%) | 58 (66.7%) |  |  |  |  |
| Pluri | 1135 (35.5%) | 29 (33.3%) |  |  |  |  |
| Conception* |  |  | 0.044 |  |  |  |
| Natural | 1972 (61.6%) | 44 (50.6%) |  |  |  |  |
| ART | 1227 (38.4%) | 43 (49.4%) |  |  |  |  |
| Mode of delivery |  |  | 0.853 |  |  |  |
| Vaginal dellivery | 283 (8.8%) | 7 (8.0%) |  |  |  |  |
| Cesarean section | 2916 (91.2%) | 80 (92.0%) |  |  |  |  |
| Height (cm) |  |  | 0.602 |  |  |  |
| < 160 | 1081 (33.8%) | 29 (33.3%) |  |  |  |  |
| 160-169 | 2006 (62.7%) | 57 (65.5%) |  |  |  |  |
| > = 170 | 112 (3.5%) | 1 (1.1%) |  |  |  |  |
| Pre-pregnancy BMI (kg/m^2^) * |  |  | 0.088 |  |  |  |
| <18.5 | 655 (21.5%) | 13 (15.1%) |  |  |  |  |
| 18.5-23.9 | 1888 (61.9%) | 65 (75.6%) |  |  |  |  |
| 24.0-27.9 | 402 (13.2%) | 7 (8.1%) |  |  |  |  |
| >=28.0 | 107 (3.5%) | 1 (1.2%) |  |  |  |  |
| HDP* |  |  | 0.167 |  |  |  |
| No | 2799 (87.5%) | 71 (81.6%) |  |  |  |  |
| GH or cHTN | 109 (3.4%) | 3 (3.4%) |  |  |  |  |
| PE | 291 (9.1%) | 13 (14.9%) |  |  |  |  |
| Placenta previa* |  |  | <0.001 |  |  |  |
| No | 3128 (97.8%) | 65 (74.7%) |  |  | Ref. |  |
| Yes | 71 (2.2%) | 22 (25.3%) |  | 5.898 | 3.130-11.113 | <0.001 |
| Placenta accrete* |  |  | <0.001 |  |  |  |
| No | 3046 (95.2%) | 55 (63.2%) |  |  | Ref. |  |
| Yes | 153 (4.8%) | 32 (36.8%) |  | 6.694 | 3.905-11.474 | <0.001 |
| Macrosomia |  |  | 0.621 |  |  |  |
| No | 3164 (98.9%) | 86 (98.9%) |  |  |  |  |
| Yes | 35 (1.1%) | 1 (1.1%) |  |  |  |  |

*Factors assigned to multivariate logistic regression analysis.

Abbreviations: Ref., reference; PPH, postpartum hemorrhage; ART, assistant reproductive technology; BMI, body mass index; HDP, hypertensive disorders of pregnancy; cHTN, chronic hypertension; GH, gestational hypertension; PE, preeclampsia.
