# Supplementary material for: Incidence and Risk Factors of Postpartum Hemorrhage in China: A Multicenter Retrospective Study
Source: Front Med (Lausanne). 2021 Aug 23;8:673500. doi: 10.3389/fmed.2021.673500 (PMC8419315; doi:10.3389/fmed.2021.673500)
Supplement: Supplementary file 4 [file Table_4.DOCX]

Table S4. Logistics regression to identify potential risk factors for PPH in cesarean section (N = 45455).

| Variables | Group Control  (n = 45455) | Group PPH  (n = 637) | P  value | Multivariate logistic regression | | |
| --- | --- | --- | --- | --- | --- | --- |
|  |  |  |  | Adjusted OR | 95% CI | P value |
| Age(y)* |  |  | 0.002 |  |  |  |
| <25 | 1762 (3.9%) | 28 (4.4%) |  |  |  |  |
| 25-34 | 30153 (67.3%) | 387 (60.8%) |  |  |  |  |
| >=35 | 12903 (28.8%) | 222 (34.9%) |  |  |  |  |
| Parity* |  |  | <0.001 |  |  |  |
| Nulli | 21802 (48.6%) | 217 (34.1%) |  |  | Ref. |  |
| Pluri | 23016 (51.4%) | 420 (65.9%) |  | 1.562 | 1.298-1.878 | <0.001 |
| Conception* |  |  | <0.001 |  |  |  |
| Natural | 42324 (94.4%) | 574 (90.1%) |  |  |  |  |
| ART | 2494 (5.6%) | 63 (9.9%) |  |  |  |  |
| Group gestation* |  |  | <0.001 |  |  |  |
| Singleton | 41902 (93.5%) | 557 (87.4%) |  |  | Ref. |  |
| Twin | 2916 (6.5%) | 80 (12.6%) |  | 3.227 | 2.451-4.249 | <0.001 |
| Height (cm) |  |  | 0.370 |  |  |  |
| < 160 | 16184 (36.1%) | 239 (37.5%) |  |  |  |  |
| 160-169 | 27005 (60.3%) | 381 (59.8%) |  |  |  |  |
| > = 170 | 1629 (3.6%) | 17 (2.7%) |  |  |  |  |
| Pre-pregnancy BMI (kg/m^2^) * |  |  | <0.001 |  |  | 0.003 |
| <18.5 | 9224 (21.3%) | 95 (15.2%) |  | 0.868 | 0.680-1.109 | 0.258 |
| 18.5-23.9 | 26749 (61.8%) | 387 (62.1%) |  |  | Ref. |  |
| 24.0-27.9 | 5737 (13.3%) | 111 (17.8%) |  | 1.301 | 1.024-1.654 | 0.031 |
| >=28.0 | 1544 (3.6%) | 30 (4.8%) |  | 1.794 | 1.176-2.736 | 0.007 |
| HDP |  |  | 0.543 |  |  |  |
| No | 40963 (91.4%) | 590 (92.6%) |  |  |  |  |
| GH or cHTN | 1206 (2.7%) | 14 (2.2%) |  |  |  |  |
| PE | 2649 (5.9%) | 33 (5.2%) |  |  |  |  |
| Placenta previa* |  |  | <0.001 |  |  |  |
| No | 42913 (95.7%) | 261 (41.0%) |  |  | Ref. |  |
| Yes | 1905 (4.3%) | 376 (59.0%) |  | 12.332 | 10.048-15.135 | <0.001 |
| Placenta accrete* |  |  | <0.001 |  |  |  |
| No | 42940 (95.8%) | 272 (42.7%) |  |  | Ref. |  |
| Yes | 1878 (4.2%) | 365 (57.3%) |  | 9.573 | 7.822-11.717 | <0.001 |
| Macrosomia |  |  | 0.434 |  |  |  |
| No | 41694 (93.0%) | 598 (93.9%) |  |  |  |  |
| Yes | 3124 (7.0%) | 39 (6.1%) |  |  |  |  |

*Factors assigned to multivariate logistic regression analysis.

Abbreviations: Ref., reference; PPH, postpartum hemorrhage; ART, assistant reproductive technology; BMI, body mass index; HDP, hypertensive disorders of pregnancy; cHTN, chronic hypertension; GH, gestational hypertension; PE, preeclampsia.
