# Supplementary material for: Incidence and Risk Factors of Postpartum Hemorrhage in China: A Multicenter Retrospective Study
Source: Front Med (Lausanne). 2021 Aug 23;8:673500. doi: 10.3389/fmed.2021.673500 (PMC8419315; doi:10.3389/fmed.2021.673500)
Supplement: Supplementary file 5 [file Table_5.DOCX]

Table S5. Logistics regression to identify potential risk factors for PPH in vaginal delivery (N = 53798).

| Variables | Group Control  (n = 53631) | Group PPH  (n = 167) | P  value | Multivariate logistic regression | | |
| --- | --- | --- | --- | --- | --- | --- |
|  |  |  |  | Adjusted OR | 95% CI | P value |
| Age(y)* |  |  | 0.072 |  |  |  |
| <25 | 4577 (8.5%) | 6 (3.6%) |  |  |  |  |
| 25-34 | 41679 (77.7%) | 137 (82.0%) |  |  |  |  |
| >=35 | 7375 (13.8%) | 24 (14.4%) |  |  |  |  |
| Parity* |  |  | 0.047 |  |  |  |
| Nulli | 36192 (67.5%) | 125 (74.9%) |  |  | Ref. |  |
| Pluri | 17439 (32.5%) | 42 (25.1%) |  | 0.647 | 0.452-0.925 | 0.017 |
| Conception* |  |  | <0.001 |  |  |  |
| Natural | 52838 (98.5%) | 157 (94.0%) |  |  |  |  |
| ART | 793 (1.5%) | 10 (6.0%) |  |  |  |  |
| Group gestation* |  |  | <0.001 |  |  |  |
| Singleton | 53348 (99.5%) | 160 (95.8%) |  |  | Ref. |  |
| Twin | 283 (0.5%) | 7 (4.2%) |  | 6.682 | 2.902-15.388 | <0.001 |
| Height (cm) |  |  | 0.467 |  |  |  |
| < 160 | 16723 (63.5%) | 112 (67.1%) |  |  |  |  |
| 160-169 | 34037 (31.2%) | 49 (29.3%) |  |  |  |  |
| > = 170 | 2871 (5.4%) | 6 (3.6%) |  |  |  |  |
| Pre-pregnancy BMI (kg/m^2^) * |  |  | 0.039 |  |  |  |
| <18.5 | 13335 (25.4%) | 33 (20.1%) |  |  |  |  |
| 18.5-23.9 | 33843 (64.4%) | 106 (64.6%) |  |  |  |  |
| 24.0-27.9 | 4560 (8.7%) | 19 (11.6%) |  |  |  |  |
| >=28.0 | 782 (1.5%) | 6 (3.7%) |  |  |  |  |
| HDP * |  |  | 0.001 |  |  | 0.002 |
| No | 52450 (97.8%) | 155 (92.8%) |  |  | Ref. |  |
| GH or cHTN | 777 (1.4%) | 6 (3.6%) |  | 2.429 | 1.058-5.580 | 0.036 |
| PE | 404 (0.8%) | 6 (3.6%) |  | 3.866 | 1.575-9.485 | 0.003 |
| Placenta previa * |  |  | <0.001 |  |  |  |
| No | 53408 (99.6%) | 153 (91.6%) |  |  | Ref. |  |
| Yes | 223 (0.4%) | 14 (8.4%) |  | 20.367 | 11.236-36.918 | <0.001 |
| Placenta accreta * |  |  | <0.001 |  |  |  |
| No | 53038 (98.9%) | 147 (88.0%) |  |  | Ref. |  |
| Yes | 593 (1.1%) | 20 (12.0%) |  | 10.674 | 6.504-17.518 | <0.001 |
| Macrosomia * |  |  | 0.001 |  |  |  |
| No | 51207 (95.5%) | 149 (89.2%) |  |  | Ref. |  |
| Yes | 2424 (4.5%) | 18 (10.8%) |  | 2.737 | 1.662-4.506 | <0.001 |

*Factors assigned to multivariate logistic regression analysis.

Abbreviations: Ref., reference; PPH, postpartum hemorrhage; ART, assistant reproductive technology; BMI, body mass index; HDP, hypertensive disorders of pregnancy; cHTN, chronic hypertension; GH, gestational hypertension; PE, preeclampsia.
