# Supplementary material for: Incidence and Risk Factors of Postpartum Hemorrhage in China: A Multicenter Retrospective Study
Source: Front Med (Lausanne). 2021 Aug 23;8:673500. doi: 10.3389/fmed.2021.673500 (PMC8419315; doi:10.3389/fmed.2021.673500)
Supplement: Supplementary file 6 [file Table_6.docx]

Table S6. The impact of parity on PPH in the overall population and the four subgroups.

|  | P  value | Multivariate logistic regression | | |
| --- | --- | --- | --- | --- |
|  |  | Adjusted OR | 95% CI | P value |
| Overall population (N = 99253) | <0.001 |  |  | 0.002 |
| Nulli |  |  | Ref. |  |
| Pluri (1-3) |  | 1.288 | 1.099-1.509 | 0.002 |
| Pluri (≥4) |  | 2.175 | 1.018-4.651 | 0.045 |
| Singletons (N = 95967) | <0.001 |  |  |  |
| Nulli |  |  | Ref. |  |
| Pluri (1-3) |  | 1.383 | 1.170-1.635 | < 0.001 |
| Pluri (≥4) |  | 1.968 | 0.789-4.905 | 0.146 |
| Twins (N = 3286) | 0.157 |  |  | NS |
| Nulli |  |  | Ref. |  |
| Pluri (1-3) |  |  | NA |  |
| Pluri (≥4) |  |  | NA |  |
| Cesarean delivery (N = 45455) | <0.001 |  |  |  |
| Nulli |  |  | Ref. |  |
| Pluri (1-3) |  | 1.542 | 1.281-1.857 | <0.001 |
| Pluri (≥4) |  | 2.979 | 1.347-6.590 | 0.007 |
| Vaginal delivery (N = 53798) | 0.136 |  |  |  |
| Nulli |  |  | Ref. |  |
| Pluri (1-3) |  | 0.655 | 0.458-0.937 | 0.021 |
| Pluri (≥4) |  | NA | NA | NA |

Abbreviations: Ref., reference; PPH, postpartum hemorrhage; NS, not significant; NA, not applicable.
